# Investigating the Role of the Catalyst within Resorcinol–Formaldehyde Gel Synthesis

**DOI:** 10.3390/gels7030142

**Published:** 2021-09-15

**Authors:** Elisha Martin, Martin Prostredny, Ashleigh Fletcher

**Affiliations:** Department of Chemical and Process Engineering, University of Strathclyde, Glasgow G1 1XJ, UK; elisha.martin@strath.ac.uk (E.M.); martin.prostredny@strath.ac.uk (M.P.)

**Keywords:** resorcinol–formaldehyde, xerogels, aerogels, porous, sol–gel, pH

## Abstract

Resorcinol–formaldehyde (RF) gels are porous materials synthesized via a sol–gel reaction and subsequently dried, producing structures with high surface areas and low densities—properties that are highly attractive for use in various applications. The RF gel reaction takes place in the presence of a catalyst, either acidic or basic in nature, the concentration of which significantly impacts final gel properties. The full extent of the catalyst’s role, however, has been subject to debate, with the general consensus within the field being that it is simply a pH-adjuster. The work presented here explores this theory, in addition to other theories postulated in the literature, through the synthesis and analysis of RF gels catalysed by mixtures of relevant compounds with varying concentrations. The relationship between catalyst concentration and initial solution pH is decoupled, and the individual roles of both the cation and the anion within the catalyst are investigated. The results presented here point towards the significance of the metal cation within the RF gel reaction, with similar structural properties observed for gels synthesized at constant Na^+^ concentrations, regardless of the initial solution pH. Furthermore, through the use of alternative cations and anions within catalyst compounds, the potential effects of ions on the stabilization of macromolecules in solution are explored, the results of which suggest a ‘Hofmeister-like’ series could be applicable within the catalysis of RF gel reactions.

## 1. Introduction

### 1.1. Resorcinol–Formaldehyde Gels

Owing to their highly attractive and tuneable properties, resorcinol–formaldehyde (RF) gels have been the focus of numerous studies over the years [1,2,3,4]. Their synthesis procedure, as established by Pekala [5], involves a sol–gel reaction, a subsequent solvent exchange step, and, lastly, a drying process to produce the lightweight, porous structure of the final gel. Attempts to optimize this synthesis process have included explorations of gelation temperature [6] and drying methods [7], as well as extending to investigations into material doping [8,9]. Furthermore, the computational modelling of RF gel formation and analysis has also been explored, progressing towards efficient computational tailoring of their properties [10,11,12].

Although widely accepted within the field, the ‘catalyst’ used in the RF sol–gel reaction cannot technically be considered a catalyst, given that it is used up and cannot be recovered at the end of the process. Still, the term ‘catalyst’ remains commonly used in this context and will, therefore, be used in the work discussed here. Either an acidic or basic catalyst can be used within RF gel synthesis, each increasing the rate of reaction through different mechanisms. In the case where the RF reaction takes place in the presence of a basic catalyst, the addition reaction begins with the abstraction of a proton from the resorcinol molecule, forming an anion. This abstraction subsequently leads to increased reactivity of the resorcinol molecules. The addition reaction proceeds, typically with molecules of formaldehyde positioning themselves at two available carbon atoms on the benzene ring, with the resulting molecules being known as hydroxymethyl derivatives. This addition reaction, under basic catalysis, is fast, producing numerous hydroxymethyl derivatives, which react to form many small clusters. After this point, a condensation reaction proceeds, the hydroxymethyl derivatives releasing H_2_O as they form bridged structures, linking with other hydroxymethyl groups or with unreacted resorcinol molecules. The condensation step takes place slowly under basic catalysis; however, this step takes place quickly under acid catalysis, with the acid increasing the rate of reaction through protonation of hydroxymethyl derivatives, producing structurally different materials. The pH of the catalyst used, therefore, is crucial to the properties of the materials formed. However, research conducted over the years has indicated that its role may be far more complex. Elucidating the full extent of this role could provide invaluable insight into the RF gel formation mechanism, as well as opening new avenues for material tailoring to achieve a wide range of structural properties.

### 1.2. The Role of pH

When Pekala first established the process of synthesizing RF gels, he made use of a basic catalyst, sodium carbonate (Na_2_CO_3_), which has remained as the most common catalyst used for RF gel formation. As previously mentioned, acid catalysts can also be utilized, in addition to other basic compounds such as potassium hydroxide [13], lithium carbonate [14], and sodium hydrogen carbonate [15], amongst others. The nature of the catalyst, being acidic or basic, in addition to its concentration, will impact the initial pH value of the RF solution, consequently impacting the rate at which the resorcinol–formaldehyde reaction takes place and, therefore, impacting the final structural properties. The role of the catalyst beyond this has been subject to some debate, where many works have concluded that the pH-determining impact of the anion (CO_3_^2−^ in the case of sodium carbonate) is the predominant role, if not the sole role. Other works, meanwhile, have explored the impact of the cation (Na^+^ in the case of sodium carbonate), concluding that it is also significant in determining final gel properties, and that its role cannot be discounted. Direct comparisons between research presented in the literature is, however, very complex, given that the wide variety of synthesis conditions used within each study leads to the formation of materials with markedly different textural properties, some of which result in the observation of different trends.

In an attempt to understand the role of pH alone, research was carried out by Lin and Ritter [16] which made use of dilute nitric acid to adjust the initial solution pH of sodium carbonate-catalysed RF gels. This work involved the synthesis of R/C50 gels with initial solutions containing 5 *w*/*v*% solids, which then underwent acetone solvent exchange before ambient drying, followed by gel pyrolysis. Nitrogen adsorption analysis of the final gels revealed a clear trend—as the initial solution pH incrementally decreased from pH 7 to pH 6, the resulting gel surface area increased significantly. Gels produced from initial solution pH values above 7 were found to yield almost no surface area, whilst those produced from pH values between 5.5 and 6 achieved equally high surface areas. This work highlights the crucial role of pH in the RF gel reaction, where the condensation reaction rate is increased by the presence of additional H^+^ ions, resulting in the formation of increasingly cross-linked structures. Despite this, the specific roles of the catalyst components were not explored in this work, and new components were included in the system through the addition of nitric acid. This could have implications on the final gel structure, including through the potential acid–base reaction that may have taken place, with the addition of HNO_3_ resulting in decreased concentration of carbonate, given that HNO_3_ is a stronger acid and carbonic acid is unstable. In the cases where an acidic catalyst alone is used, studies have found that the initial mixture must possess a pH within the range of 1–4 in order to produce a gel with a viable structure, albeit with visual and textural properties that are distinct from those of base-catalysed reactions [17]. Finally, initial solution pH values below 1 have been shown to result in precipitation, while pH values within the range of 4–5.5 have been found to be insufficient for the successful catalysis of either the addition or the condensation reactions taking place, the resulting product being a non-porous powder rather than an interlinked porous structure [14]. From experiments carried out within our research group, the pH of an initial RF gel solution with no catalyst added whatsoever is 4.1, which, as expected, fails to produce inter-connected porous materials.

The role of pH was explored further by Job et al. [18], who synthesized RF gels using sodium hydroxide solution to achieve set initial solution pH values ranging from 5.45 to 7.35. For gels analysed after vacuum drying, as solution pH increased from 5.45 to 6.5, the total surface area also increased from 330 to 510 m^2^/g, with a decrease observed after this point at pH 7.35. Given that the increasing solution pH corresponds to increasing Na^+^ concentration from the addition of sodium hydroxide solution, the role of Na^+^ could be considered in this work, however, Job et al. conclude that the cation present in the standard Na_2_CO_3_ catalyst plays no direct role whatsoever and that pH alone is the determining factor in final gel properties. Subsequent studies have affirmed pH-altering as the catalyst’s sole purpose, this becoming the general consensus within the field, with suggestions that the same effects from pH alterations could be achieved using any base that does not react with resorcinol or formaldehyde [2,19].

### 1.3. The Role of Individual Catalyst Components

A small number of studies have attempted to look beyond the pH-adjusting role of the catalyst, and instead focus on the role of its components in greater detail. One such study by Horikawa et al. [15] investigated the impacts of changing both the anion and cation present, comparing the textural properties of pyrolyzed RF aerogels catalysed by Na_2_CO_3_, K_2_CO_3_, NaHCO_3_, and KHCO_3_ at R/C50. Nitrogen adsorption analysis of the resulting gels produced isotherm profiles for each, the visual inspection of which can provide insight into both the size and type of pores present. The results revealed clear similarities, with Na_2_CO_3_ and K_2_CO_3_ gels producing visually comparable isotherms, suggesting that their structural properties were also comparable. The isotherm profiles of NaHCO_3_ and KHCO_3_ were also visually similar, indicating that the impact of changing cation from Na^+^ to K^+^ is negligible. The change in structural properties when the anion was changed from CO_3_^2−^ to HCO_3_^−^ were evident, including in the isotherm profile, the total pore volume, and the average pore width observed across the four gel samples, with HCO_3_^−^ gels possessing larger pores with a greater total pore volume in comparison to those of the CO_3_^2−^ gels. In this case, an additional complexity is involved, given that changing the anion from CO_3_^2−^ to HCO_3_^−^ also halves the concentration of metal cations present, making the specific role of each difficult to ascertain.

A study carried out by Calvo et al. [20] took a different approach to investigating the role of the catalyst in the RF reaction, this time producing microwave-synthesized xerogels catalysed by five different compounds—Na_2_CO_3_, Li_2_CO_3_, NaHCO_3_, Ca(OH)_2_, and NaOH. In this work, as opposed to synthesizing gels at a set R/C ratio, the catalyst was added until the desired pH was reached, therefore the mass of catalyst added depended on its alkalinity. No definitive trends were observed for variations in the cation used, however, clear porosity differences were evident for anion variations. Gels synthesized using hydroxide catalysts (Ca(OH)_2_, and NaOH) possessed smaller pores, also with narrower pore size distributions, in comparison with those synthesized by carbonate catalysts. The total mesopore volumes of the gels were also significantly reduced, with hydroxide catalysed gels possessing mesopore volumes approximately one-third of those observed for gels produced from carbonate catalysts. Calvo et al. suggest that this could be attributable to the size of the anions used, with the larger CO_3_^−^ ions causing steric hindrances and leading to the formation of wider pores. The conclusion drawn was that the role of the anion within the catalyst is far more significant than that of the cation; however, the same complexity discussed previously still applies. Although the pH values of the initial solutions are equal for each gel synthesized in this study, the concentration of both cations and anions vary depending on the catalyst alkalinity, therefore, elucidating their individual roles is more complex.

Further research was carried out by Job et al. [21] where RF xerogels were prepared with six different catalysts—LiOH, NaOH, KOH, Ca(OH)_2_, Ba(OH)_2_, and Sr(OH)_2_—once again with different masses added until the desired pH was reached. Their findings revealed distinct differences in the properties of gels formed by alkali metal hydroxides (LiOH, NaOH, and KOH, each possessing cations of M+ charge) in comparison to those of alkaline earth metal hydroxides (Ca(OH)_2_, Ba(OH)_2_, and Sr(OH)_2_), each possessing cations of M^2+^ charge), with the two groups producing structures with average pore widths in the ranges of 50–80 nm and 70–100 nm, respectively. Taylor et al. [14,22] investigated the specific role of the cation further, this time using RF xerogels synthesized using four alkali metal carbonates—Li_2_CO_3_, Na_2_CO_3_, K_2_CO_3_, and Cs_2_CO_3_—all prepared at equal R/C ratios, alongside their subsequent work, which included alkaline earth metal carbonates, CaCO_3_ and BaCO_3_. Once again, gels synthesized using alkaline earth metal carbonates, possessing M^2+^ cations as opposed to M^+^ within alkali metal carbonates, comprised of pores much larger in diameter, alongside an increase in total pore volume, indicating that the role of the cation present could be significant.

Earlier research carried out by Grenier-Loustalot et al. [23] investigated the impact of the valency and ionic radius of the metal cation used within the catalyst for the formation of phenol–formaldehyde (PF) gels. This study found that the use of divalent cations resulted in an increased rate of formaldehyde consumption during the PF reaction in comparison to monovalent species, as did the use of cations with larger ionic radii, allowing the authors to conclude that the nature of the cation plays an important role in the reaction kinetics. The study proposed a mechanism by which this could takes place, where the metal cation participates in establishing an intermediate chelated molecule during the phenol–formaldehyde addition reaction, which could also be applicable for RF reactions.

The studies discussed here, which often produce variable or conflicting results, demonstrate the complexity of the RF gel formation mechanism, and the difficultly with which many research groups have attempted to determine the role of the catalyst within the RF reaction. The work carried out to date has provided valuable insight into the different parameters influencing the RF reaction, however, the roles of the individual catalyst components are still yet to be fully understood, particularly given the difficultly in decoupling their relationship with one another and with the resulting pH.

### 1.4. Ionic Solution Effects—The Hofmeister Series

Through the work carried out by Taylor et al. [14,22], another theory emerged suggesting that the ions present within the catalyst could contribute to RF gel formation based on their ability to ‘salt-in’ or ‘salt-out’ macromolecules from solution. This is comparable to the Hofmeister series, which was established in 1888 by Franz Hofmeister [24], and which arranges ions based on the stability or instability they create for proteins in solution.

Given that the RF gel catalysts studied comprise ionic compounds, in addition to the pivotal role solubility plays in the sol–gel process, these potential effects could be an important consideration. As the mass of macromolecules increases with cluster growth, in addition to their increased cross-linking, the resulting solubility decreases, eventually reaching the point of gelation where a solid interlinked structure is formed. Investigating how different parameters impact the solubility of macromolecules, such as those observed in the RF reaction, has been the focus of various studies over the years [25,26,27]. Amongst the theories postulated, the kosmotropic and chaotropic effects of ions is of particular interest. Kosmotropes are compounds that promote the stability and rigidity of macromolecules, stabilising their intramolecular interactions, and facilitating the formation of ordered structures [28]. Chaotropes, on the other hand, contribute to the destabilising and disordering of macromolecules, disrupting non-covalent interactions, and hindering the formation of stable structures [29].

**Figure 1:**
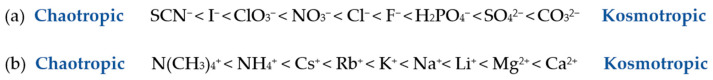
(**a**) Reversed Hofmeister series for anions, and (**b**) Hofmeister series for cations [30,31].

As previously mentioned, the Hofmeister series was created with respect to the impact of ions on proteins in solution. For hydrophilic surfaces such as RF gels, the effects of anions have shown to be reversed as a result of the ion–surface interactions taking place, and the anion Hofmeister series is reversed to reflect this [30], as shown in Figure 1a, while the Hofmeister series for cations is shown in Figure 1b. The traditional catalyst of sodium carbonate (NaCO_3_), therefore, comprises a cation with a medium kosmotropic effect and an anion with a significant kosmotropic effect. Investigating the impact of various cations and anions within the RF reaction catalysis, particularly with respect to their positioning within the Hofmeister series, could provide a deeper understanding of their role.

## 2. Results and Discussion

### 2.1. Varying Catalyst Concentration

**Figure 2:**
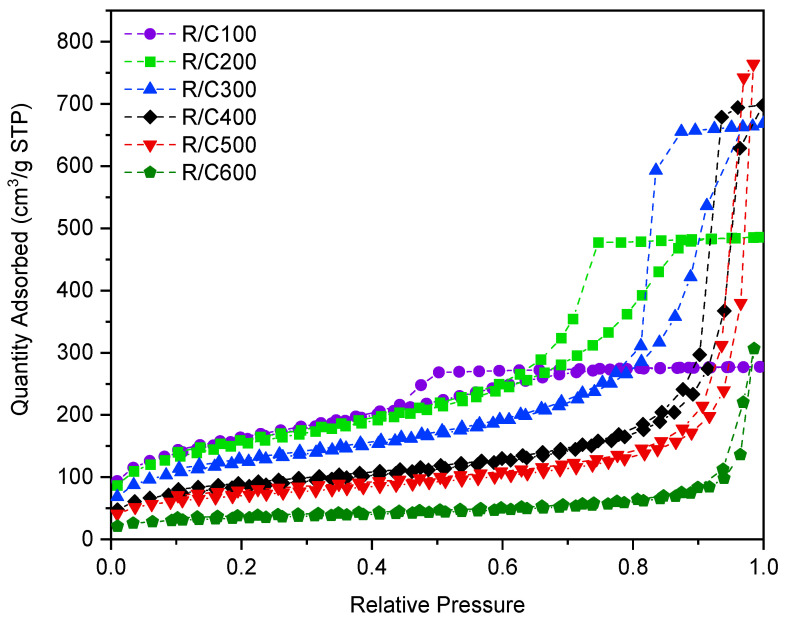
Nitrogen adsorption isotherms for sodium carbonate gels at resorcinol/catalyst molar ratios (R/C ratios) 100–600. STP abbreviates standard temperature and pressure.

**Figure 3:**
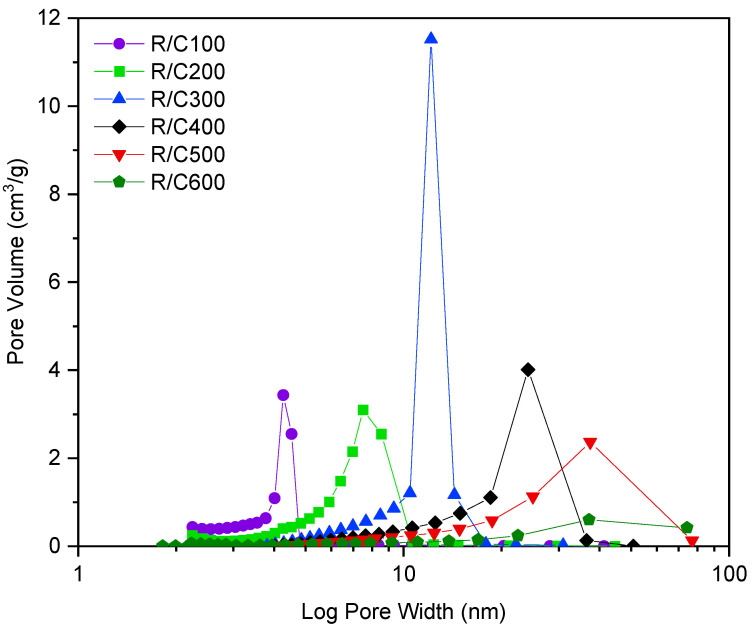
Pore size distributions for sodium carbonate gels at resorcinol/catalyst molar ratios (R/C ratios) 100–600.

The effect of varying the molar ratio of resorcinol to catalyst, referred to as R/C ratio, has been studied previously and is pivotal in determining the final textural properties of the RF gel. ‘Standard’ RF gels were prepared using Na_2_CO_3_ as the catalyst, with the R/C ratios studied being 100, 200, 300, 400, 500, and 600. Samples of each gel were analysed using nitrogen adsorption, the resulting isotherms from which are displayed in Figure 2. The isotherms shown vary significantly with changes in R/C ratio, with differing gradients of initial uptake observed, as well as hysteresis loops occurring at varying relative pressure values. As can be seen from the plots, an increase in R/C ratio corresponds to a reduced uptake at lower pressures, suggesting that fewer micropores are present, given that micropores are known to fill at lower relative pressures. Instead, the relative increase in uptake at higher pressures for lower R/C ratio materials points towards the increased presence of mesopores within these materials. Furthermore, as the R/C ratio is increased, a shift in the position of the hysteresis loop is observed, with pores filling and emptying at higher pressures, once again pointing towards the presence of larger pores, which fill at higher relative pressures. Using the data from the isotherm, the pore size distribution can be calculated using the BJH method [32], allowing further characterization of the gels’ porous structure through more than simple isotherm interpretation. Figure 3 displays the pore size distribution for the standard RF gels at R/C ratios of 100 to 600, where the *y*-axis is the pore volume corresponding to each pore width on the *x*-axis, with the total area below each plot being representative of the total pore volume of the analysed gel. Table 1 details the average pore width value of each of the gels studied, in addition to its BET [33] surface area.

As indicated by the isotherms for the standard gels (Figure 2), an increase in R/C ratio is thought to lead to an increase in average pore width. The graph in Figure 3 confirms this, displaying the pore size distribution for each sample visibly shifting towards larger pore width values, as the R/C ratio increases. The increase in pore size associated with high R/C ratios is thought to be as a result of the reduced number of initial clusters formed, leading to the growth of larger clusters over time, the process of which occurs at a slower rate [14]. These larger clusters subsequently have larger pores between them, impacting the textural properties and overall appearance of the resulting gels. The higher concentration of catalyst present in gels synthesized using lower R/C ratios results in a higher number of initial clusters formed, the total mass therefore being distributed over a greater number of smaller clusters, the process of which takes place more rapidly. Given that the clusters formed are of smaller average size, they subsequently have smaller pores between them, once again impacting the textural properties and overall appearance of the gels formed [14]. The gels formed at low R/C ratios, therefore, possess a higher degree of porosity, with a greater number of pores of smaller widths, and an increased surface area per gram, making them useful for applications such as filters, sorbing media for waste containment, or hydrogen fuel storage [34].

### 2.2. Investigating the Role of the Cation

As has been previously discussed, various research has been published on the impact of catalyst concentration on the final gel properties, with most referring to the catalyst purely as a means to alter the pH of the initial solution, labelling it simply as a ‘pH adjuster’. Whilst the impact of initial solution pH is undeniably fundamental to the final textural properties of the gel, as outlined above, some studies have indicated that the role of the catalyst components extends far beyond this. The main hurdle in fully understanding the role of the catalyst is in attempting to decouple the relationship between the concentration of catalyst components present and the resulting pH of the gel solution, given that one cannot be altered without affecting the other. It is, of course, possible to keep the catalyst concentration constant whilst adjusting the pH through additions of acidic or basic solutions, however, this method introduces new additional components into the gel solution, which could ultimately alter the final structure of the gel formed, potentially as a result of ‘salting-out’ effects previously discussed. With this in mind, the research presented here aims to decouple the relationship between pH and the catalyst concentration, in addition to analysing the role of the specific components of the catalyst used. Firstly, the role of the cation used within the catalyst was assessed through the preparation and analysis of different sets of gel samples with the following catalyst combinations:

1(i) Na_2_CO_3_/NaHCO_3_ mixture at constant cation concentrations equivalent to R/C100

1(ii) Na_2_CO_3_/NaHCO_3_ mixture at constant cation concentrations equivalent to R/C300

2(i) NaHCO_3_/NH_4_HCO_3_ mixture at constant anion concentrations equivalent to R/C100

2(ii) NaHCO_3_/NH_4_HCO_3_ mixture at constant anion concentrations equivalent to R/C300

Using these combinations of catalyst species, the specific roles of the cation and anion can be investigated, with sample sets 1(i) and 1(ii) exploring the effects of varying solution pH with the addition of H^+^ ions while the Na^+^ concentration remains constant. Sample sets 2(i) and 2(ii), on the other hand, vary the concentration of Na^+^ ions while the concentration of HCO_3_^−^ remains constant, providing further insight into the significance of the role of the metal cation. The mass of catalyst required for each combination was calculated based on the percentage of total sodium ions (Na^+^) contributed by the two catalysts being compared (in the cases where Na_2_CO_3_ and NaHCO_3_ mixtures are used), or the percentage of total hydrogen carbonate ions (HCO_3_^−^) contributed by the two catalysts being compared (in the cases where NaHCO_3_ and NH_4_HCO_3_ mixtures are used). In samples labelled 25Na_2_:75NaH, this refers to a sample where 25% of the total moles of Na^+^ present are contributed by Na_2_CO_3_, whilst 75% are contributed by NaHCO_3_. Similarly, in samples labelled 25NaH:75NH_4_, this refers to a sample where 25% of the total moles of HCO_3_^−^ present are contributed by NaHCO_3_, whilst 75% are contributed by NH_4_HCO_3_. All samples within a series such as this maintain a constant concentration of Na^+^ or HCO_3_^−^ ions equivalent to that of a standard gel at a given R/C ratio, but with varying contribution percentages from the two catalysts.

#### 2.2.1. Catalyst Mixtures—Na_2_CO_3_/NaHCO_3_

**Figure 4:**
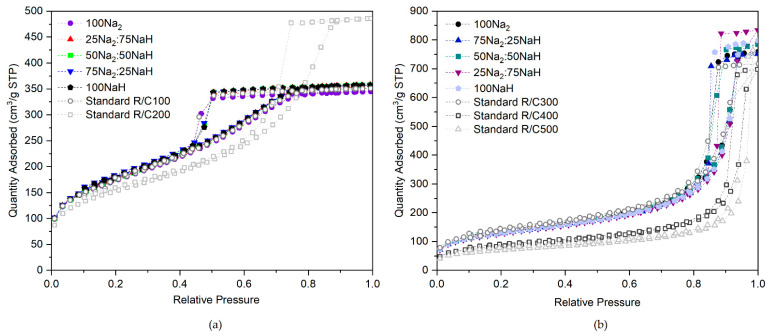
(**a**) Isotherms from sample set 1(i) Na_2_/NaH R/C100 Na^+^ equivalent, (**b**) sample set 1(ii) Na_2_/NaH R/C300 Na^+^ equivalent. Note that R/C is the resorcinol/catalyst molar ratio.

First, consider the isotherms of sets 1(i) and (ii), shown in Figure 4a,b, where the Na^+^ concentration remains constant and equal to that of standard gels prepared at R/C ratios of 100 and 300, respectively. Despite the variations in initial solution pH as the concentration of H^+^ ions present increases through the addition of NaHCO_3_, which will be discussed in more detail below, the resulting isotherms remain similar in shape. They each follow the same trend throughout the adsorption process and exhibit similar hysteresis loops, which also occur at very similar partial pressure values. Minute variations in hysteresis loops, such as those observed, are to be expected between gel samples that are similar in makeup and structure. Furthermore, not only are the isotherms within sets 1(i) and 1(ii) indistinguishable from one another within experimental error, but they are also comparable to those obtained for standard gels prepared at the same equivalent R/C ratio in terms of Na^+^ concentrations. This suggests that the Na^+^ ion is one of the central components in determining the properties of the final material, especially given that the varying concentrations of anions present in the different catalyst mixtures appear to have negligible effects. It is also worth noting that the gels labelled 100NaH, synthesized using 100% NaHCO_3_ as the catalyst to provide Na^+^ concentrations equal to R/C100 and R/C300 standard Na_2_CO_3_ gels, are actually representative of R/C50 and R/C150 gels with respect to the ratio of resorcinol to NaHCO_3_ catalyst concentration.

#### 2.2.2. Catalyst Mixtures—NaHCO_3_/NH_4_HCO_3_

**Figure 5:**
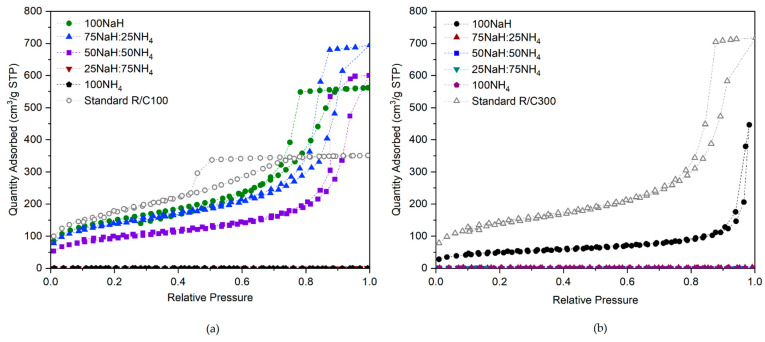
Isotherms for (**a**) sample set 2(i) NaH/NH_4_ R/C100 HCO_3_^−^ equivalent, and (**b**) sample set 2(ii) NaH/NH_4_ R/C300 HCO_3_^−^ equivalent. Note that R/C is the resorcinol/catalyst molar ratio.

Next, consider the isotherms for sets 2(i) and (ii) displayed in Figure 5a,b, where the HCO_3_^−^ concentration remains constant and equal to that of standard gels prepared at R/C ratios of 100 and 300, respectively. The isotherms within the R/C100 set (Figure 5a) show a gradual change as the Na^+^ concentration is reduced and replaced by NH_4_^+^ until 75% of the HCO_3_^−^ ions present are contributed by NH_4_HCO_3_, at which point the resulting materials prove to be non-porous. The isotherms shown within Figure 5b indicate that a porous material was formed only when 100% of the HCO_3_^−^ ions were sourced from NaHCO_3_ at R/C300, with any substitution for NH_4_HCO_3_ producing a non-porous material. When the samples are then compared to those of standard gels prepared at the same equivalent R/C ratio, the isotherms are different again, even varying from those prepared with 100% NaHCO_3_. This could be explained by the fact that the R/C ratio for this sample set is made on the basis of equivalency in anion concentration, therefore resulting in half the Na^+^ concentration when NaHCO_3_ is used in comparison to Na_2_CO_3_. Note that the initial solutions for all gels in sample sets 2(i) and 2(ii) fell within the required pH window for the successful formation of porous structures previously discussed, with pH values across the samples ranging between 6 and 7. Importantly, the visual differences displayed here between the isotherms of 100% NaHCO_3_ R/C100 gels and standard Na_2_CO_3_ R/C100 gels are in strong agreement with those observed in the work carried out by Horikawa et al. [15], discussed previously, who used nitrogen adsorption measurements to determine the comparative properties of pyrolyzed RF aerogels catalysed by Na_2_CO_3_, K_2_CO_3_, NaHCO_3_, and KHCO_3_, each at R/C50.

In this work, the cations used within the catalyst are Na^+^ and NH_4_^+^, where the substitution of one for the other achieved significantly different results. The presence of Na^+^ ions appeared to aid the gelation process, leading to the formation of inter-linked porous network structures, while the presence of NH_4_^+^ failed to promote the gelation process, with its sole use leading to the formation of a non-porous material that had failed to gel completely. As postulated by Taylor et al. [22], the ability of these ions to ‘salt-in’ or ‘salt-out’ macromolecules from solution, as with the Hofmeister series, could be an important factor within the results observed. In accordance with the reverse Hofmeister series suggested for hydrophilic surfaces, shown in Figure 1a, Na^+^ ions have medium kosmotropic effects, therefore promoting the stability of macromolecules in solution, and facilitating the formation of inter-linked porous structures in solution [28]. NH_4_^+^ ions, on the other hand, have chaotropic effects, therefore contributing to the destabilizing and disordering of macromolecules, and hindering the formation of porous structures in solution [29]. This could have important implications on the final properties of the structures, with the potential precipitation of solids from solution taking place early in the presence of chaotropic ions (such as NH_4_^+^), resulting either in the slower growth of the clusters which remain in the solution, or significant precipitation hindering the formation of inter-linked porous structures. Furthermore, if the particles within the solution become too large without interconnections, the system is no longer colloidal, and the large particles will sediment.

These results show promise in elucidating the role of the metal ion, indicating that the concentration of Na^+^ present from the catalyst is pivotal in determining the gelation process and subsequent structural properties of the final material.

### 2.3. Structural Impacts of Initial Solution pH

The results obtained from sample sets 1(i) and 1(ii) can be used to investigate the role of the metal ion in comparison to that of the measured initial solution pH, aiming to decouple the overall relationship between catalyst concentration and pH. Referring back to Figure 4, which displays the isotherms from sets 1(i) and 1(ii) alongside isotherms for standard gels across comparable initial solution pH ranges, these can be considered in addition to the resulting gel textural properties from the analysis shown in Table 2.

**Figure 6:**
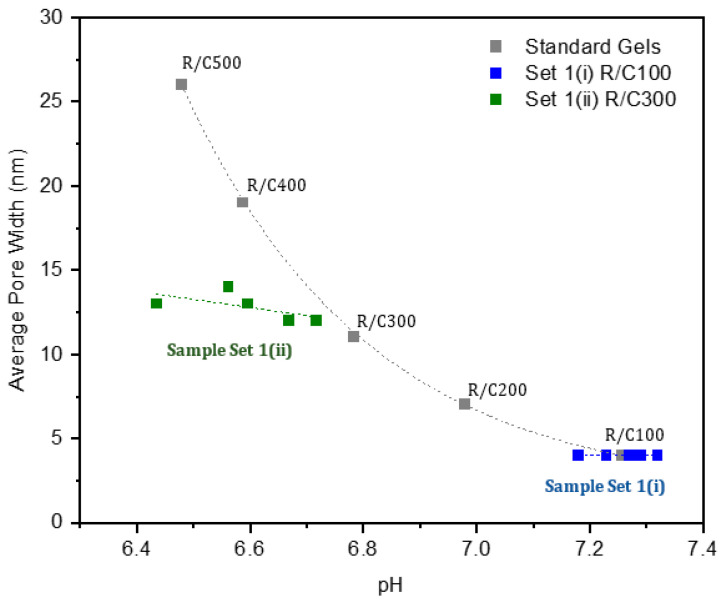
Graph of initial solution pH versus average pore width for the three sets of gels. Note that for sample sets 1(i) and 1(ii), the points marked on the graph show increasing NaH concentration from right to left. Original R/C100–R/C500 gels are plotted with a dashed line according to exponential decay curve fitting, while a straight-line fit is used for sample sets 1(i) and 1(ii). R/C is the resorcinol/catalyst molar ratio.

Within sample sets 1(i) and 1(ii), a gradient of decreasing initial solution pH is observed, with the concentration of H^+^ ions increasing as Na_2_CO_3_ is substituted for NaHCO_3_. Using this method to alter the pH introduces no new components into the system, limiting the number of variables being altered at a given time, and therefore allowing a more accurate analysis to be carried out. Figure 6 shows graphically how the average pore widths of the gels vary with pH for three sets of samples: sample sets 1(i), 1(ii), and also standard Na_2_CO_3_ gels at RC ratios 100–500, while Table 2 indicates the initial pH of the prepared solutions during gel synthesis for each of the three sets of gels alongside the average pore width determined from nitrogen adsorption analysis of the resulting gels.

As the data in Table 2 shows, the average pore width within the standard Na_2_CO_3_ gel structure is significantly impacted by the corresponding R/C ratio used during its synthesis, with average widths ranging from 4–26 nm across an R/C ratio range of 100–500. Conversely, on inspection of the average pore widths within sample sets 1(i) and 1(ii), very little variation is observed, despite the change in initial solution pH. Furthermore, looking at sample set 1(ii) specifically, the initial solution pH of the gels ranges from 6.44–6.72. This range is similar to that observed across standard Na_2_CO_3_ gels at R/C ratios 300–500, which possess initial pH values of 6.48–6.78. Despite the similar range of initial pH values observed across the two sets, the final gels formed are markedly different. Samples in set 1(ii), which each possess the same concentration of Na^+^ ions, all have average pore widths of around 12–14 nm. This is in sharp contrast to that of the standard Na_2_CO_3_ gels, which each have different concentrations of Na^+^ ions, and whose average pore widths vary significantly from 11–26 nm. The isotherms in Figure 4, discussed previously, further highlight the differences in the structures formed, providing a comparison of the adsorption behaviour of the sample within sets 1(i) and 1(ii) with those of standard Na_2_CO_3_ gels which possess a similar pH range.

Although pH undoubtedly plays an important role in the catalysis of the RF reaction and impacts the textural properties of the final gel when significant pH adjustments are made, this is likely not observed with relatively small changes in pH such as those in this experiment (<0.4), provided the solution pH falls within the viable range of 5.5–7 for gels made with a basic catalyst. Instead, the variations observed in gel properties within the pH ranges studied suggests that the influence of pH alone may have previously been overestimated, and points towards the pivotal role of the cation in the formation of the porous structure of the gel. This could be particularly relevant to the work carried out by Job et al. [18], discussed previously, where RF gels were synthesized at set pH values, achieved through the addition of NaOH solution. In this work, as the pH increased from 5.45 to 7.35, corresponding to the increasing volume of NaOH added, the average pore size consistently decreased from 50 nm to 4 nm. Although the authors concluded that the change in pH was responsible for the significant differences in textural properties achieved, the results presented here suggest that those differences could, in fact, be attributed to the increasing Na^+^ concentration, as larger volumes of NaOH solution was added.

### 2.4. Investigating the Role of the Anion

In order to further investigate the role of the anion within the catalyst, two other sodium salts, containing alternative anions that do not hydrolyse, thus should not change the solution pH, were added as catalysts during RF gel synthesis. Sodium chloride (NaCl) and sodium sulphate (Na_2_SO_4_) were chosen in this work and the combined amounts of both salts were based on molar ratio of sodium ions to resorcinol molecules in the solution, so that the total amount of sodium ions was kept constant and equal to the amount in solution for an RF gel prepared using only sodium carbonate and R/C100. For example, a sample of Na_2_CO_3_ 200 NaCl 100 was made using the amount of Na_2_CO_3_ as for a standard gel with R/C200 and amount of NaCl to adjust the sodium ion concentration in the reaction solution to match that of a standard gel solution for R/C100, the numbers used in the sample name represent the individual R/C ratios for the salts used. When a gel was made without sodium carbonate, its R/C ratio was labelled as Na_2_CO_3_ INF.

**Figure 7:**
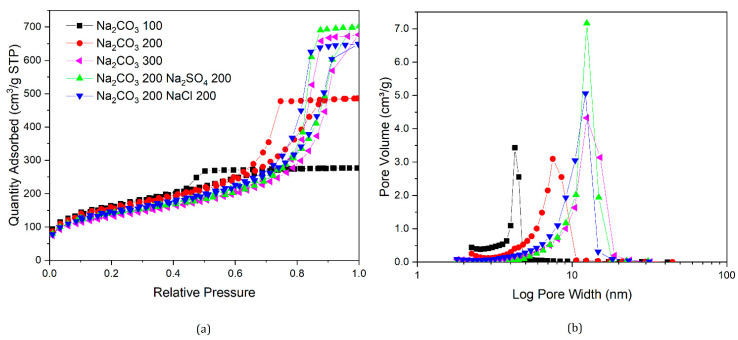
Nitrogen sorption isotherms of gels with varying catalyst compositions (**a**), and corresponding pore size distributions (**b**).

**Figure 8:**
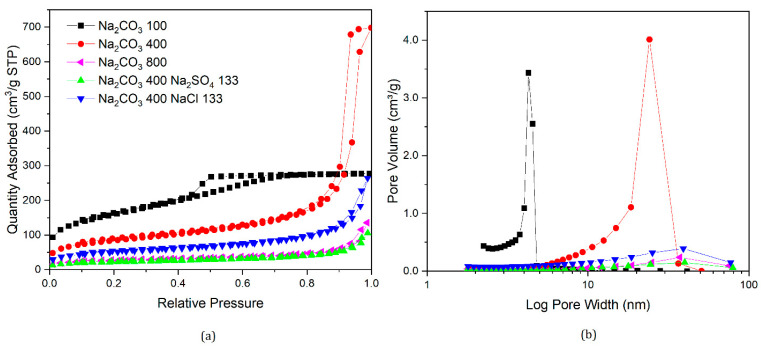
Nitrogen sorption isotherms of gels with modified catalyst composition (**a**) and corresponding pore size distributions (**b**).

**Figure 9:**
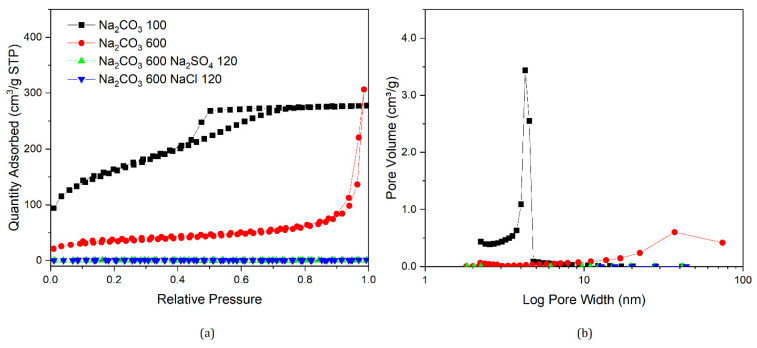
Nitrogen sorption isotherms of gels with modified catalyst composition (**a**) and corresponding pore size distributions (**b**).

**Figure 10:**
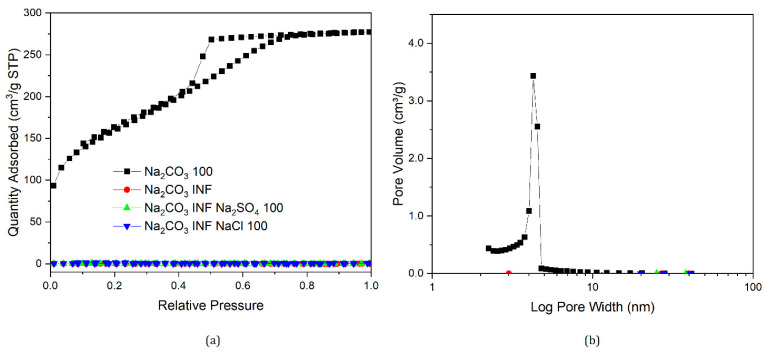
Nitrogen sorption isotherms of gels with modified catalyst composition (**a**) and corresponding pore size distributions (**b**). Note that INF represents infinite resorcinol/catalyst molar ratio (R/C ratio); therefore, zero concentration.

Nitrogen sorption isotherms and their corresponding pore size distributions for samples prepared in this section are presented in Figure 7, Figure 8, Figure 9 and Figure 10, along with data for selected samples prepared with only sodium carbonate. It can be seen from Figure 7 that both nitrogen sorption isotherms and pore size distributions for gels made with Na_2_CO_3_ 200 and added NaCl or Na_2_SO_4_ exhibit similar shapes to that of a standard gel with R/C300, rather than R/C100 or 200. The solutions for samples with varying catalyst compositions had the same sodium ion concentration as a sample with R/C100, so if sodium ion concentration was the main factor influencing the final structure, these should exhibit similar textural properties. However, the solution pH would differ from a standard R/C100 solution, since both chloride and sulphate anions do not hydrolyse in an aqueous solution resulting in a similar pH to that of a R/C200 solution. However, the pH values presented in Table 3 show a slight difference between the solutions with and without the additional salts present. It should be noted that the pH probe used converts a voltage measurement into a pH value, which means the voltage reading could be influenced by the other ions present in the solution. Nevertheless, there is a general trend of decreasing pH with increasing R/C ratio, as a result of decreasing concentration of hydrolysing carbonate ions. Due to the strong electrolyte nature of Na_2_CO_3_, its dissociation in an aqueous solution should not be affected by the addition of Na^+^ ions.

Similar trends can be observed in Figure 8 and Figure 9 where the nitrogen sorption data for samples with additional salts are closer to samples prepared with a higher R/C ratio than the ones with the same Na_2_CO_3_ amount or sodium ion concentration. Samples with Na_2_CO_3_ 400 exhibit similar properties to a standard gel with R/C ratio 800. Interestingly, when either Na_2_SO_4_ or NaCl are added to a gel with Na_2_CO_3_ 600, a non-porous material is obtained, similar to when no catalyst is used, even though RF gels prepared using only Na_2_CO_3_ at R/C ratio 600 are still porous materials.

According to the work by Taylor et al. [14,22], discussed previously, dynamic light scattering (DLS) experiments showed that RF clusters that are formed in the reaction solution gradually grow and adhere to each other, resulting in the final structures observed in the dried materials. Textural properties of RF xerogels, therefore, depend on the final cluster size and their packing in three dimensions. The anions used in this part of the study are arranged in the reversed Hofmeister series [30], as presented in Section 1.3 for hydrophilic surfaces, in the following order of ability to salt-out macromolecules from solution: CO_3_^2−^ < SO_4_^2−^ < Cl^−^. This suggests, that in the solution containing only the carbonate (CO_3_^2−^) ions, the growing clusters will precipitate from the solution at later stages compared with solutions, where a proportion of the carbonate ions are substituted by anions with higher salting-out ability, such as sulphate (SO_4_^2−^) and chloride (Cl^−^) anions.

The gelation mechanism of RF gels has been investigated by dynamic light scattering (DLS) by Taylor et al. [14], providing new insights into the proposed cluster formation and growth process. As clusters grow in the reaction solution, they can potentially grow at slightly different rates, based on the diffusion of reagents towards the reactive centres. When a cluster reaches critical size, leading to local phase separation, the newly created interface will lead to adsorption of species dissolved in the solution, reagents, and on this interface. In a solution containing ions with a higher salting-out ability (SO_4_^2−^ and Cl^−^), clusters might precipitate at an earlier stage, leading to a faster subsequent growth of these clusters due to the reagent adsorption effects. This could lead to the clusters left in solution growing at a slower pace, with the reactants being depleted by the faster growing precipitated clusters. This might result in an increased final size of clusters in from these solutions, compared to when only CO_3_^2−^ is present. Without the SO_4_^2−^ and Cl^−^ ions, a larger number of clusters grow at a similar rate for longer time, with larger clusters present when phase separations occur, leading to more uniform and, on average, smaller particles present in the final material. The larger clusters, arising from an earlier phase separation, would have larger gaps in between them, observed as larger pore sizes from nitrogen sorption measurements. However, it is important to keep in mind that only pores up to the upper limit of mesopores, up to ∼50–100 nm [35,36,37], are observable by this technique, with the macropores not filling, and thus, not contributing towards the average pore size.

A series of RF gels using only sodium chloride as a catalyst were prepared, in order to investigate whether the presence of sodium ions is the major driving force for RF gel formation rather than solution pH. Samples with NaCl R/C 12.5, 200, and 400 (corresponding to sodium ion concentrations for Na_2_CO_3_ R/C ratios of 25, 400, and 800) were prepared; however, all of the final materials were found to be non-porous. The values of solution pH, after all the reagents had dissolved and a 30-min stirring period, were measured as 3.40, 3.30, and 3.24 for NaCl R/C 12.5, 200, and 400, respectively. This, in addition to the results presented previously, suggests that both the presence of sodium ions and appropriate solution pH value are necessary in order to obtain a viable porous gel structure. It can, therefore, be concluded that both the cation and the anion within the catalyst play a central role, the potential ‘salting-in’ or ‘salting-out’ effect of both being pivotal to the structural properties of the final gel formed, in addition to the required solution pH window discussed previously.

**Figure 11:**
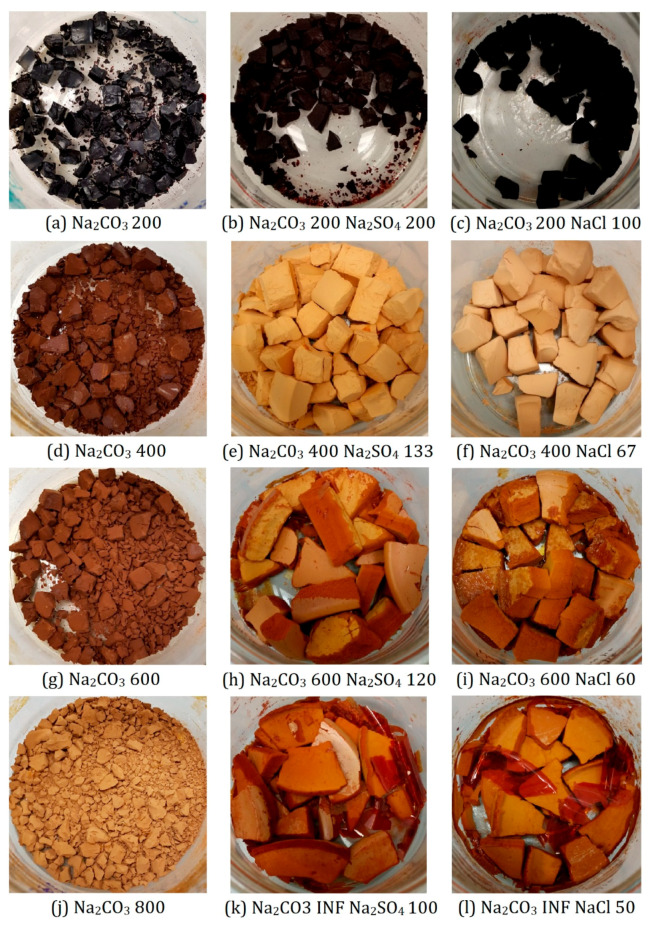
RF xerogels prepared with varying R/C rations of Na_2_CO_3_, Na_2_SO_4_, and NaCl.

An interesting observation can be made by visually comparing dried RF xerogels made with and without the additional salts, photographs of these xerogels are presented in Figure 11. Xerogels prepared with either sodium sulphate or sodium chloride added have a very similar appearance and differ significantly from the standard xerogels prepared with sodium carbonate only. It is also worth mentioning that samples with Na_2_CO_3_ R/C ratios 400 and 600 made with additional salts did not exhibit the usual level of shrinkage after subcritical drying, as all the other studied materials do, even though they were all cut into similar sized pieces (~1 cm), which can be used as a visual guide in comparing samples, prior to the solvent exchange step. This might be explained by the larger pore sizes present in these samples, eliminating capillary forces during drying while preserving the extent of cross-linking, leading to a lower degree of material shrinkage. If large macropores are present in these samples, as described above, the liquid–vapor interface would not cause a collapse of this large-scale porous structure, leading to only a small shrinkage of these materials even under subcritical conditions. However, it is important to note that for many applications, such as gas storage, this macroporosity is not as useful as the presence of mesopores or micropores.

## 3. Conclusions

The role of the individual catalyst components within the RF gelation reaction was investigated, the results of which point towards a pivotal role beyond simply just pH-adjusting—the theory that is generally accepted within the RF gel research field. Gels prepared at a constant hydrogen carbonate concentration with varying sodium ion concentrations were shown to vary significantly, some failing to gel whatsoever. Conversely, gels prepared with a constant sodium ion concentration with varying carbonate concentrations all possessed similar textural properties, despite their differences in initial solution pH. These results confirm the significance of the metal cation in the gel synthesis and allow its role to be decoupled from the role of pH, which is generally thought to be the most crucial factor in the gelation mechanism.

Furthermore, replacing a proportion of the sodium carbonate catalyst with sodium chloride or sodium sulphate leads to materials with significantly different textural properties. The introduction of chloride or sulphate ions into the reaction solution appeared to have a similar effect to that observed for increasing sodium carbonate R/C ratio (decreasing catalyst concentration), suggesting that the presence of these ions has a comparatively adverse effect on gelation. Given that the addition of sodium chloride and sodium sulphate increased the concentration of sodium ions present, yet still had this adverse effect on gelation, the ability of ions to ‘salt-in’ or ‘salt-out’ macromolecules from solution was considered. A Hofmeister-like series for the RF gel reaction could, therefore, be possible. In the case of sodium sulphate and sodium chloride, the cation has strong ‘salting-in’ capabilities while the anions have weaker ‘salting-in’ capabilities, or even strong ‘salting-out’ capabilities in comparison the carbonate anion typically used. This affects the point at which the aggregating clusters precipitate from solution, resulting in gels that possess different structural properties to those catalysed using sodium carbonate alone.

## 4. Materials and Methods

### 4.1. RF Gel Synthesis

A series of RF gels were synthesised with varying compositions, each gel formed through an established procedure requiring four reagents: resorcinol (ReagentPlus, 99%, Sigma-Aldrich, Poole, UK), formalin solution (37 wt % formaldehyde in water and methanol, Sigma-Aldrich, Poole, UK), deionised water (produced in-house with Millipore Elix 5, Progard 2), and a catalyst. In this work, the catalysts used were sodium carbonate (Na_2_CO_3_, anhydrous, ≥99.5%, Sigma-Aldrich, Poole, UK), sodium hydrogen carbonate (NaHCO_3_, anhydrous, ≥99.5%, Sigma-Aldrich, Poole, UK), ammonium hydrogen carbonate (NH_4_HCO_3_, 99%, Fisher Scientific, Loughborough, UK), sodium sulphate (Na_2_SO_4_, anhydrous, ≥99.0%, Sigma-Aldrich, Poole, UK), and sodium chloride (NaCl, Redi-Dri, anhydrous, ≥99%, Sigma-Aldrich, Poole, UK).

In the preparation of the gels investigated here, the volume of liquid added per gel in the initial solution was kept constant at 60 mL, which included the water and methanol content of the formalin solution. The total solids content of the initial mixture was kept constant at 12 g, which included the mass of formaldehyde contained within the formalin solution. The mass of resorcinol (R), formaldehyde (F), and catalyst (C) was varied according to the R/C ratio being prepared, all the while maintaining a constant R/F ratio of 0.5 in accordance with the stoichiometry of the resorcinol–formaldehyde reaction. Data for the mass of individual components of each reagent are included within the Supplementary Materials, in addition to details of how the volume of formalin required for each gel was calculated.

The RF gel synthesis follows a standard procedure, with the reagents initially combined in individual circular glass jars according to the desired catalyst concentration and mixture, forming the initial RF solution. For gels synthesized using a combination of two catalysts, the two compounds were weighed into separate crucibles and added to the mixture simultaneously. The resulting solution pH was measured using a Hanna Instruments benchtop pH meter (Leighton Buzzard, Bedfordshire, UK), after which point the jar was sealed and placed into a Memmert ULE-500 oven (Büchenbach, Germany) at 85 °C and left to gel for a 3-day period.

After the gelation period was complete, the gels underwent a 3-day solvent exchange process, where the water within the pores was exchanged for acetone. Acetone was selected as a solvent for exchange, due to its low surface tension value (23.46 mN/m for acetone in comparison to 71.99 mN/m for water; both values taken at 25 °C [38]), therefore, minimizing the extent of pore shrinkage upon drying, in addition to its miscibility with water.

Finally, following the completion of the 3-day solvent exchange step, the jars containing RF gel samples were drained, covered with perforated aluminum foil, and placed into a Towson and Mercer 1425 Digital Vacuum Oven (Stretford, UK). After closing the vacuum oven door, the oven heating was turned on and the temperature was set to 110 °C, which corresponds to 85 ± 5 °C inside the oven (monitored using a thermometer placed inside the oven). The vacuum pump (Vacuubrand MZ 2C NT, Wertheim, Germany) attached to the oven was turned on, with two solvent traps with water/ice mixture placed between the oven and the pump, to condense acetone evaporating from the gel samples. The solvent traps were used to preserve the vacuum pump, limiting the amount of solvent vapor that came into contact with the membranes, as well as monitoring sample drying. For safety purposes, the oven and the pump were not left running overnight but were turned on at the beginning of each working day for 8 h. The gels were dried for 2 days, after which they were transferred into labelled sample containers.

### 4.2. Nitrogen Sorption Measurements

Nitrogen sorption measurements were carried out to determine the structural properties of the final gels, again following a standard procedure. Once the RF gels had been dried, a sample of approximately 0.5 g was weighed into a bulb tube, initially undergoing a degassing process using a Micromeritics ASAP 2420 surface area and porosity analyser (Hexton, UK). Following this, the sample was transferred to an analysis port within the equipment and the nitrogen sorption measurements were carried out. The analysis lasted for approximately 20–30 h per sample, collecting 40 data points for adsorption as the relative pressure was incrementally increased from 0.1 to 1 and then 30 data points for desorption as the relative pressure was decreased from 1 to 0.1. Subsequent analysis of the isotherm data included surface area determination using BET theory [33], and the Rouquerol correction for microporous samples [35]. Where calculations of total pore volume and micropore volume were carried out, the t-plot method [39] was employed, and finally, average pore size determination using the Barrett–Joyner–Halenda (BJH) method [32]. The BJH method was used to determine the pore size distribution within the meso- and macroporous range, assuming pores of cylindrical shape were present, applying data taken from the desorption branch of the isotherm. The principle of the BJH method relies on the calculation of the Kelvin core radius at each relative pressure interval:

(1) $$\ln\left(\frac{p}{p_{o}}\right)=\frac{2\gamma V_{m}}{r_{c}RT}$$

where $p/p_{o}$ is relative pressure, $r_{c}$ is pore meniscus radius, γ is the surface tension of the liquid–vapor interface, $V_{m}$ is the liquid molar volume, R is the universal gas constant and T is temperature. The total pore radius is composed of the meniscus radius ($r_{c}$) in addition to the thickness of the remaining layer adsorbed onto the pore walls (t). The BJH method, therefore, includes the calculation of this thickness in order to calculate the total pore width, and for calculations applicable to nitrogen adsorption onto RF gels, an empirical formula known as the carbon black equation [40] was used:

(2) $$t=2.98+6.45\left(\frac{p}{p_{o}}\right)+0.88{\left(\frac{p}{p_{o}}\right)}^{2}$$

## Figures and Tables

**Table 1:** The corresponding average pore widths and Brunauer-Emmet-Teller (BET) surface areas. Values are reported to an accuracy less than their error; therefore, their error values have been omitted. Note that S_BET_ is BET surface area, $\bar{\phi }$ is average pore width, and R/C ratio is the molar ratio of resorcinol to catalyst.

R/C Ratio	$\bar{\phi }$ (nm)	S_BET_ (m^2^/g)
100	4	574
200	7	552
300	11	446
400	19	304
500	26	254
600	28	124

**Table 2:** Initial pH values of standard gels at varying resorcinol/catalyst molar ratios (R/C ratios), in addition to that of samples in sets 1(i) and 1(ii), and their corresponding average pore widths. The average pore width values are recorded to an accuracy less than their error, therefore their error values have been omitted. Note that $\bar{\phi }$ is average pore width.

Standard Gels	pH	$\bar{\phi }$ (nm)
R/C100	7.26	4
R/C200	6.98	7
R/C300	6.78	11
R/C400	6.59	19
R/C500	6.48	26
**Sample Set 1(i) R/C100**	**pH**	$\bar{\phi }$ **(nm)**
100Na_2_	7.32	4
75Na_2_:25NaH	7.29	4
50Na_2_:50NaH	7.27	4
25Na_2_:75NaH	7.23	4
100NaH	7.18	4
**Sample Set 1(ii) R/C300**	**pH**	$\bar{\phi }$ **(nm)**
100Na_2_	6.72	12
75Na_2_:5NaH	6.67	12
50Na_2_:50NaH	6.59	13
25Na_2_:75NaH	6.56	14
100NaH	6.44	13

**Table 3:** pH values of initial solutions and textural properties for gels with different resorcinol/catalyst molar ratios (R/C ratios) of sodium carbonate (Na_2_CO_3_), sodium sulphate (Na_2_SO_4_), and sodium chloride (NaCl). Note that S_BET_ is Brunauer-Emmet-Teller (BET) surface area, V_T_ is total pore volume, V_u_ is micropore volume, and $\bar{\phi }$ is average pore width.

R/C Ratio			pH	S_BET_ (m^2^/g)	V_T_ (cm^3^/g)	V_u_ (cm^3^/g)	$\bar{\phi }$ (nm)
Na_2_CO_3_	Na_2_SO_4_	NaCl					
100	-	-	7.26	574	0.43	0.06	4
200	-	-	6.98	552	0.75	0.06	7
400	-	-	6.59	304	1.08	0.04	19
600	-	-	6.27	124	0.30	0.02	28
-	-	-	4.1	-	-	-	-
200	200	-	7.4	490	0.95	0.06	9
200	-	100	7.3	500	0.99	0.06	10
400	133	-	7.0	120	0.30	0.02	16
400	-	67	6.9	130	0.34	0.02	17
600	120	-	6.7	1	-	-	34
600	-	60	6.6	-	-	-	39
-	100	-	3.3	-	-	-	-
-	-	50	3.1	-	-	-	-

## Data Availability

The data presented in this study are openly available in the University of Strathclyde KnowledgeBase Pure Portal, https://doi.org/10.15129/17dfa992-9818-42d6-8afe-20da1ece457d (accessed on 7 September 2021).

## Supplementary Materials

Data for the mass of individual components of each reagent used within gel synthesis is available online at https://www.mdpi.com/article/10.3390/gels7030142/s1, Table S1: Initial Solution Composition for Na_2_CO_3_ Gels, Table S2: Initial Solution Composition for Na2CO3/NaHCO3 Mixture Gels—R/C100 Equivalent, Table S3: Initial Solution Composition for Na2CO3/NaHCO3 Mixture Gels—R/C300 Equivalent, Table S4: Initial Solution Composition for NaHCO3/NH4CO3 Mixture Gels—R/C100 Equivalent, Table S5: Initial Solution Composition for NaHCO3/NH4CO3 Mixture Gels—R/C300 Equivalent, Table S6: RF gel solution compositions for study of sodium chloride as additional source of sodium ions. Numbers in sample name correspond to R/C ratios of catalyst salts, INF represents infinite R/C ratio (zero concentration), and Table S7: RF gel solution compositions for study of sodium sulphate as additional source of sodium ions. Numbers in sample name correspond to R/C ratios of catalyst salts, INF represents infinite R/C ratio (zero concentration).
