# Supplementary material for: Investigating the Role of the Catalyst within Resorcinol–Formaldehyde Gel Synthesis
Source: Gels. 2021 Sep 15;7(3):142. doi: 10.3390/gels7030142 (PMC8482184; doi:10.3390/gels7030142)
Supplement: Supplementary file 1 [file gels-07-00142-s001.zip › gels-1368678-supplementary.pdf]

gels

MDPI

Supplementary Materials

Investigating the Role of the Catalyst Within Resorcinol–Formaldehyde Gel Synthesis

Elisha Martin, Martin Prostredny and Ashleigh Fletcher *

Department of Chemical and Process Engineering, University of Strathclyde, Glasgow G1 1XJ, UK; elisha.martin@strath.ac.uk (E.M.); martin.prostredny@strath.ac.uk (M.P.)
* Correspondence: ashleigh.fletcher@strath.ac.uk; Tel.: +44-(0)141-548-2431

Gel Synthesis Data for the Investigation of the Cation Role

Table S1. Initial Solution Composition for Na_2CO_3 Gels. R/C ratio is the resorcinol/catalyst molar ratio.

R/C Ratio	Resorcinol (g)	Formaldehyde (g)	Na_2CO_3 (g)
**100**	7.7170	4.2087	0.07428
**200**	7.7409	4.3318	0.03726
**300**	7.7490	4.2262	0.02486
**400**	7.7530	4.2284	0.01866
**500**	7.7554	4.2297	0.01493
**600**	7.7570	4.2306	0.01244

Table S2. Initial Solution Composition for Na_2CO_3/$NaHCO_3$ Mixture Gels—R/C100 Equivalent. R/C ratio is the resorcinol/catalyst molar ratio.

Na_2:NaH Na^+ Source Ratio	Resorcinol (g)	Formaldehyde (g)	Na_2CO_3 (g)	$NaHCO_3$ (g)
**100:0**	7.7170	4.2087	0.07428	-
**75:25**	7.7030	4.2049	0.05566	0.02941
**50:50**	7.6961	4.2011	0.03707	0.05877
**25:75**	7.6961	4.1973	0.01852	0.08807
**0:100**	7.6891	4.1935	-	0.11733

Table S3. Initial Solution Composition for Na_2CO_3/$NaHCO_3$ Mixture Gels—R/C300 Equivalent. R/C ratio is the resorcinol/catalyst molar ratio.

Na_2:NaH Na^+ Source Ratio	Resorcinol (g)	Formaldehyde (g)	Na_2CO_3 (g)	$NaHCO_3$ (g)
**100:0**	7.7490	4.2262	0.02486	-
**75:25**	7.7466	4.2249	0.01864	0.00985
**50:50**	7.7443	4.2236	0.01242	0.01969
**25:75**	7.7419	4.2223	0.00621	0.02953
**0:100**	7.7396	4.2211	-	0.03937

Table S4. Initial Solution Composition for $NaHCO_3/NH_4CO_3$ Mixture Gels—R/C100 Equivalent. R/C ratio is the resorcinol/catalyst molar ratio.

NaH:NH4 HCO_3^- Source Ratio	Resorcinol (g)	Formaldehyde (g)	$NaHCO_3$ (g)	NH_4HCO_3 (g)
**100:0**	7.7269	4.2141	0.05895	-
**75:25**	7.7275	4.2144	0.04422	0.01387
**50:50**	7.7280	4.2148	0.02948	0.02774
**25:75**	7.7286	4.2151	0.01474	0.04162
**0:100**	7.7291	4.2154	-	0.05549

Table S5. Initial Solution Composition for $NaHCO_3/NH_4CO_3$ Mixture Gels—R/C300 Equivalent. R/C ratio is the resorcinol/catalyst molar ratio.

NaH:NH4 HCO_3^- Source Ratio	Resorcinol (g)	Formaldehyde (g)	$NaHCO_3$ (g)	NH_4HCO_3 (g)
**100:0**	7.7523	4.2280	0.01972	-
**75:25**	7.7525	4.2281	0.01479	0.00464
**50:50**	7.7527	4.2282	0.00986	0.00928
**25:75**	7.7529	4.2283	0.00493	0.01392
**0:100**	7.7530	4.2284	-	0.01855

Gel Synthesis Data for the Investigation of the Anion Role

Table S6. RF gel solution compositions for study of sodium chloride as additional source of sodium ions. Numbers in sample name correspond to R/C ratios of catalyst salts, INF represents infinite R/C ratio (zero concentration). R/C ratio is the resorcinol/catalyst molar ratio.

Sample Name	Resorcinol (g)	Formaldehyde (g)	Na_2CO_3 (g)	NaCl (g)
**Na_2CO_3 200 NaCl 200**	7.7409	4.2218	0.03726	0.04083
**Na_2CO_3 400 NaCl 133**	7.7530	4.2284	0.01866	0.06134
**Na_2CO_3 600 NaCl 120**	7.7570	4.2306	0.01244	0.06819
**Na_2CO_3 INF NaCl 100**	7.7651	4.2349	-	0.08191

Table S7. RF gel solution compositions for study of sodium sulphate as additional source of sodium ions. numbers in sample name correspond to R/C ratios of catalyst salts, INF represents infinite R/C ratio (zero concentration). R/C ratio is the resorcinol/catalyst molar ratio.

Sample Name	Resorcinol (g)	Formaldehyde (g)	Na_2CO_3 (g)	Na_2SO_4 (g)
**Na_2CO_3 200 Na_2SO_4 200**	7.7089	4.2043	0.03710	0.04972
**Na_2CO_3 400 Na_2SO_4 133**	7.7048	4.2021	0.01854	0.07454
**Na_2CO_3 600 Na_2SO_4 120**	7.7035	4.2014	0.01236	0.08281
**Na_2CO_3 INF Na_2SO_4 100**	7.7008	4.1999	-	0.09934

Additional Information

The tables above include the mass of formaldehyde (F) used within the RF gel reaction, added to the system as Formalin solution (37 wt% formaldehyde in water and methanol, Sigma-Aldrich, Poole, UK). To calculate the volume of formalin to be added to meet the required mass of F for an R/F molar ratio of 0.5 (in line with the stoichiometry of the RF reaction), the approximate mass of F per ml of Formalin was first calculated:

$$\begin{aligned} &Mass\ of\ F\ per\ Volume\ of\ Formalin\ (g/ml) \\ &= Density\ of\ Formalin\ (g/ml) \times 37\%\ (wt\%\ of\ F\ in\ Formalin) \\ &= 1.09\ g/ml \times 37\% \end{aligned}$$

$$= 0.4033\ g\ of\ F\ per\ ml\ of\ Formalin\ (g/ml)$$

The volume of Formalin added to each gel, therefore, was simply calculated from:

$$Volume\ of\ Formalin\ Required\ (ml) = \frac{Mass\ of\ F\ Required\ for\ R/F\ Molar\ Ratio\ (g)}{0.4033\ g\ of\ F\ per\ ml\ of\ Formalin\ (g/ml)}$$
